# Immunization Against *Chlamydia trachomatis* Polymorphic Membrane Protein D Tetrapeptide Motifs Limits Early Female Reproductive Tract Infection in a Mouse Model

**DOI:** 10.3390/vaccines13030234

**Published:** 2025-02-25

**Authors:** Amanda L. Collar, Andzoa N. Jamus, Julian Flanagan, Susan B. Core, William M. Geisler, Cosette M. Wheeler, Kathryn M. Frietze

**Affiliations:** 1 Department of Molecular Genetics and Microbiology, University of New Mexico Health Sciences, Albuquerque, NM 87131, USA; 2 Department of Medicine, University of Alabama at Birmingham, Birmingham, AL 35294, USA; 3 Center for HPV Prevention, University of New Mexico Comprehensive Cancer Center, University of New Mexico Health Sciences, Albuquerque, NM 87131, USA

**Keywords:** chlamydia, virus-like particle (VLP), vaccine, peptide vaccine, polymorphic membrane protein D (PmpD)

## Abstract

**Background/Objectives**: *Chlamydia trachomatis* (Ct) is a common pathogen causing urogenital, anal, oral, and ocular infections. Although extensive vaccine efforts have been underway for decades, there is no licensed vaccine available to prevent human Ct infection. Polymorphic membrane protein D (PmpD) is a highly conserved protein present on the surface of Ct elementary bodies, suggesting an important role Ct biology. Repetitive tetrapeptide motifs GGA(I,L,V) and FxxN are conserved across Pmps and are important for adhesion in the related *Chlamydia pneumoniae* Pmp21. **Methods**: Using bioinformatics approaches, we identified amino acids 270 to 294 of PmpD that included two GGA(I,L,V) motifs and an FxxN motif as vaccine targets. Synthetic peptides corresponding to these regions were chemically conjugated separately via the carboxy (C)- or amino (N)-terminus (FxxN 1.1 and FxxN 1.2) to the surface of Qβ virus-like particles (VLPs) and were tested for immunogenicity and protective capacity in mice. **Results**: Female mice immunized three times with a mixture of Qβ-FxxN 1.1 and Qβ-FxxN 1.2 vaccines without exogenous adjuvant elicited geometric-mean endpoint dilution titers near 10^4^. Further, mice showed decreased infection at early time points when challenged vaginally with luciferase-expressing *Chlamydia muridarum* over 9 days and a faster time to undetectable infection compared to controls. Immunization with individual vaccines (Qβ-FxxN 1.1 or Qβ-FxxN 1.2) did not show the same degree of reduction. **Conclusions**: Vaccination against PmpD tetrapeptide motifs is a novel and promising approach for limiting initial *Chlamydia* infection and warrants further investigation to characterize the mechanism of protection.

## 1. Introduction

*Chlamydia trachomatis* (Ct) is the cause of the most common bacterial sexually transmitted infection worldwide. Indeed, annually, there are an estimated 3.98 million new cases of Ct infection in the United States, with an estimated direct medical cost of USD 516 million [1]. This makes Ct infection the most costly non-viral STI in the United States [2]. Although Ct infection can be effectively treated via antibiotics, most women exhibit asymptomatic infection, which may go untreated without regular surveillance [3,4]. Yet, routine screening only reaches approximately half of eligible women in the United States [5]. Further, untreated or repeated Ct infection can cause serious medical sequelae in women, including pelvic inflammatory disease, ectopic pregnancy, and tubal factor infertility. Together, due to its high prevalence, the relative ineffectiveness of screening programs, and morbidity in women, there is an urgent need for an effective prophylactic Ct vaccine. Indeed, both the World Health Organization and the National Institute of Allergy and Infectious Diseases have called for increased research into areas that could benefit Ct vaccine development [6,7].

Much of the preclinical protein-based Ct vaccine efforts have focused on a single antigen: Major Outer Membrane Protein (MOMP) [8]. This is due to the immunodominant nature of MOMP. However, MOMP antibodies tend to be serotype-specific, making it difficult to protect against all Ct serovars with a single vaccine. Additionally, many preclinical vaccines targeting MOMP provide only partial protection against urogenital *Chlamydia* infections [8]. The only urogenital *Chlamydia* vaccine to be tested in humans is a subunit vaccine consisting of a concatemer of several serotype-specific peptides of MOMP corresponding to the variable domain 4 [9]. Although MOMP has been identified as a promising vaccine target, other Ct adhesion factors may provide advantages as vaccine antigens, such as providing cross-protection across Ct serovar or enhancing the protection afforded by MOMP vaccines.

**Figure 1:**
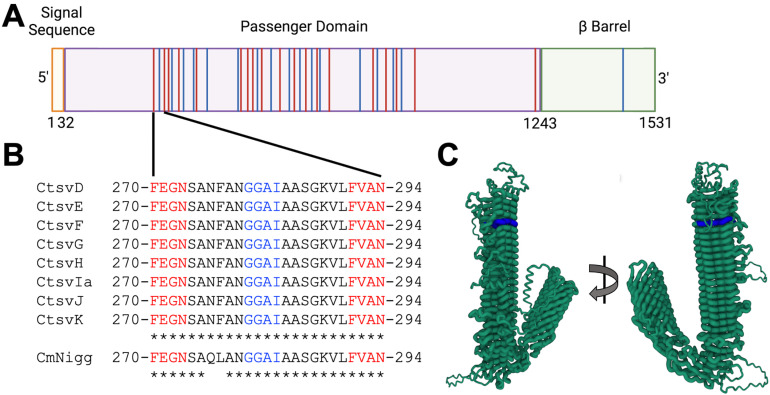
Rational design of PmpD tetrapeptide motifs for display on Qβ VLPs. (**A**) Schematic of PmpD, with the signal sequence (AA 1–32) displayed in orange, the passenger domain (AA 32–1243) displayed in purple, and the β barrel (AA 1243–1531) displayed in green. FxxN motifs are displayed in red and GGA(I,L,V) motifs are displayed in blue. (**B**) The first FxxN-GGA(I,L,V)-FxxN is shown, with FxxN in red and GGA(I,L,V) in blue. Sequences for all urogenital *Chlamydia trachomatis* (Ct) serovars along with *Chlamydia muridarum* (Cm) Nigg are aligned. * = perfectly conserved amino acids. (**C**) AlphaFold predicted structure of PmpD with FxxN-1 highlighted in blue.

The polymorphic membrane proteins (Pmps) are surface-exposed adhesion factors expressed in all *Chlamydia* species [10,11,12]. They resemble autotransporters, which are commonly found in Gram-negative bacterium, with a N-terminal signal sequence for translocation to the Ct membrane, followed by a passenger domain, and a C-terminal β-barrel domain [13,14] (Figure 1). Ct has nine Pmps, designated A–I, and together they represent 13.6% of the Ct-specific coding capacity, suggesting their importance in Ct biology [15]. Although Pmps have significant amino acid variability between one another, they all possess multiple repeats of FxxN (where x may be any amino acid) and GGA(I,L,V) tetrapeptide motifs [14,16,17]. The majority of Ct tetrapeptide motifs reside only in Pmps and are found almost exclusively within their passenger domains. GGA(I,L,V) repeats are only found within Pmps and exist in a singlet in only 10 other Ct proteins [16]. Further, FxxN is found, on average, 13.6 times per Pmp in Ct [16]. It has been hypothesized that these tetrapeptide repeats help Pmps adopt a beta-helix structure [18,19].

These repeated tetrapeptide motifs also play a critical role in Ct adhesion to host cells. The passenger domain of Pmp21 of *Chlamydia pneumoniae* (Cp), a closely related *Chlamydia* species that causes human respiratory infections, contributes to the adhesion of Cp to human HEp-2 cells in vitro. The passenger domain of Pmp21 adheres to HEp-2 cells more readily than even OmcB, another well-defined Ct adhesion factor [20]. Further, the passenger domain of Pmp21 has several independent binding domains, which are all capable of binding to human cells [20]. At least two copies of tetrapeptide motifs (two FxxN epitopes or one GGA(I,L,V) and one FxxN epitope) are required for adhesion to host cells [20]. Additionally, anti-Pmp21 serum preincubated with *Chlamydia pneumoniae* (Cp) is able to reduce infectivity by nearly 80%, and the preincubation of host cells with recombinant Pmp21 passenger domain peptide reduces the infectivity of Cp by nearly 90% [20]. These data support the importance of the tetrapeptide motifs of Pmps for adhesion to host cells and suggest that targeting these epitopes with a vaccine could be a promising approach to prevent infection.

PmpD, the Ct ortholog of Pmp21, is highly conserved among Ct serovars, with 99.2% homology, suggesting an important biological role for this adhesion factor and making it an attractive vaccine target for cross-protection to all Ct serovars [21,22]. PmpD, like Pmp21, is a strong mediator of adhesion to host cells, with PmpD null Ct strains having 70% reduced infectivity in vitro [23], and polyclonal anti-PmpD antibodies are pan-neutralizing across all urogenital Ct serovars in vitro [24]. Several groups have developed vaccines targeting PmpD [25,26,27]. Additionally, nearly all patients with a history of Ct mount a serum antibody response to one or more Pmps [28]. Further, among the Pmps, PmpD is among the top proteins to elicit these immune responses in humans. In fact, 91% of men with a history of Ct infection and 54% of adolescent women produce detectable serum PmpD antibodies [28]. However, the specificity and magnitude of this antibody response may vary between the biological sexes [28].

We hypothesized that high-titer antibody responses toward the tetrapeptide motifs of PmpD could limit initial stages of *Chlamydia* infection. In this work, we utilized a bacteriophage Qβ virus-like particle (VLP) vaccine platform to display the tetrapeptide motif epitopes at high valency. This vaccine strategy resulted in epitope-specific IgG antibodies as measured by peptide-based ELISAs. We assessed the impact of vaccination on early *Chlamydia muridarum* (Cm) infection using a previously developed luciferase-expressing Cm (Luc-Cm) [29,30,31]. Utilizing this mouse model of female reproductive tract infection with Luc-Cm, immunization with a VLP vaccine targeting three tetrapeptide motifs simultaneously resulted in reduced bacterial burden at the early stages of infection.

## 2. Materials and Methods

### 2.1. Production of Bacteriophage Qβ VLPs

Bacteriophage Qβ VLPs were produced in *Escherichia coli* as previously described [32,33,34]. *E. coli* C41 electrocompetent cells (Sigma Aldrich, Burlington, MA, USA) were transformed to encompass a plasmid expressing the Qβ coat protein under Kanamycin antibiotic selection. Qβ-expressing *E. coli* were grown at 37 °C in lysogeny broth (LB) with shaking. When the optical density (OD600, WPA Biowave CO8000 Cell Density Meter, BioChrom Ltd., Waterbeach, Cambridge, UK) reading was measured to be at 0.6–0.8, the culture was induced using 0.5 mM Isopropyl β-D-1-thiogalactopyranoside (IPTG). Induction continued for 3 h at 37 °C with shaking. Cells were then pelleted via centrifugation and frozen at −80 °C until isolation. Thawed cells were exposed to lysozyme solution incubated at 4 °C for 1 h and sonicated, and then we added 10 mg/mL DNase and 2 mM MgCl2 incubated at 37 °C for 1 h to aid in cell lysis. Afterwards, centrifugation was repeated for pellet cell debris, and supernatants were collected. Ammonium sulfate was added for 60% saturation and incubated at 4 °C overnight. Centrifugation at 10,000 RPM was conducted for 20 min at 4 °C, with supernatants discarded and pellets resuspended in 1× SCB. Centrifugation was repeated with supernatants collected a total of two times. Supernatants were frozen at −80 °C and held until size-exclusion chromatography isolation. Supernatants were centrifuged upon thawing and placed on a Sepharose column with small volume fractions collected. Fractions were run on a 1% agarose gel to identify those containing Qβ VLPs. Fractions containing Qβ VLPs were further confirmed via SDS-PAGE utilizing a positive control. Fractions containing Qβ VLPs were then combined and concentrated using 100 K Amicon Ultra Centrifugal Filters (Merck Millipore, Burlington, MA, USA). Qβ VLP stocks were then depleted of LPS using Triton X-114 phase extraction. Then, 1% *v/v* Triton X-114 was added to stocks and samples were vortexed and incubated on ice for five minutes, followed by a five-minute incubation at 37 °C. Samples were centrifuged at max speed for 1 min at 37 °C. The aqueous phase was moved to a clean tube, and the process was repeated a total of five times. Purified Qβ VLPs were again run on an SDS-PAGE Gel to determine concentration using hen’s egg lysozyme controls.

### 2.2. Production of Qβ VLP Vaccines Displaying Modified Tetrapeptide Motif Peptides

Purified Qβ VLPs were incubated with excess Succinimidyl 6-(beta-maleimidopropionamido)-hexanoate (SMPH, Thermo Fisher Scientific, Waltham, MA, USA) at 25 °C for 2 h with shaking. Excess SMPH was removed using 100 K Amicon Ultra Centrifugal Filters (Merck Millipore, Burlington, MA, USA). Qβ-SMPH was then incubated with modified peptide in excess, containing a triglycine linker and terminal cysteine (GenScript, Piscataway, NJ, USA) overnight at 4 °C. Excess peptide was removed via 100 K Amicon Ultra Centrifugal Filters (Merck Millipore, Burlington, MA, USA). Successful conjugation was confirmed via SDS-PAGE. Conjugated Qβ VLP vaccines were assumed to have approximately fifty percent loss due to multiple centrifugal filtrations and were mixed with 1× phosphate-buffered saline (PBS) pH 7.0 for a final concentration of 5 μg of VLP/mouse. Qβ VLP vaccines were stored at −80 °C until use.

### 2.3. Ethics Statement for Animal Studies

All animal studies were reviewed and approved by the Institutional Animal Care and Use Committee of the University of New Mexico School of Medicine (19-200867, 661FRIEKA18-05-01CR). Female BALB/c mice were obtained from the Jackson Laboratory (Bar Harbor, ME, USA).

### 2.4. Quantitating Antibody Responses to Cognate Synthetic Peptide

Bait-peptide ELISAs were performed as previously described [32,35]. Briefly, Immulon 2 High Binding 96-well flat-bottom plates (Thermo Fisher Scientific, Waltham, MA, USA) were used, all incubations were performed at 25 °C with shaking unless otherwise noted, and washes were performed three times using 1× PBS unless otherwise noted. Wells were coated with 0.5 μg of streptavidin (Invitrogen, Waltham, MA, USA) in 1× PBS and incubated overnight at 4 °C. Plates were washed and coated via 1 h incubation with 1 μg of Succinimidyl 6-(beta-maleimidopropionamido)-hexanoate (SMPH) in 1× PBS. Plates were washed and coated with 1 μg of synthetic modified cognate peptide (GenScript) via 2 h incubation. Plates were washed and incubated at 4 °C overnight with 0.5% dry milk in 1× PBS (blocking solution). Plates were washed and diluted serum (four-fold dilutions beginning at 1:40) in blocking solution was added. Incubation continued for 2 h. Plates were washed five times in 1× PBS and reacted with peroxidase-conjugated AffiniPure goat anti-mouse IgG secondary (Jackson ImmunoResearch, West Grove, PA, USA) at a 1:5000 dilution in blocking solution. Incubation continued for 45 min before the plate was washed five times with 1× PBS. Plates were reacted with 3,3′,5,5′-Tetramethylbenzidine (TMB, Merck Millipore, Burlington, MA, USA) for 15 min, at which point the reaction was quenched using 1% hydrochloric acid. Absorbance was read at 450 nm using accuSkan FC (Thermo Fisher Scientific, Waltham, MA, USA). Background absorbances (well containing no immune sera) were averaged, and values were subtracted from all experimental values.

### 2.5. Murine Immunization and Challenge Studies

Female BALB/c mice (n = 10/group) were immunized with their respective vaccine three times, three weeks apart, via intramuscular injection without exogenous adjuvant. One week prior to vaginal challenge, mice received 2.5 mg of medroxyprogesterone acetate subcutaneously to sync and prolong estrous cycles. Immediately preceding vaginal challenge, mice underwent retro-orbital eye bleed to collect sera to assess IgG antibody titers. Then, 2 × 10^4^ IFU of luciferase-expressing *Chlamydia muridarum* (Luc-Cm, kind gift from Dr. Guangming Zhong) in SPG was inserted into the vaginal cavity, and urogenital bacterial burden was assessed at days 3 through 9 post-infection via an in vivo imaging system (IVIS, Perkin Elmer, Waltham, MA, USA) [31]. Mice were given 0.2 mL IVISbrite D-Luciferin (40 mg/mL) intraperitoneally before imaging. Then, 25 min after administration, mice were put under gentle inhaled anesthesia and 1 min images using a Firefly probe were collected. Uniform regions of interest (ROIs) were placed on the urogenital region of the mice to detect bacterial burden via average radiance, with background average radiance subtracted from experimental values.

### 2.6. Human Samples and ELISAs

The use and collection of human serum samples was approved as previously described [35] by the University of Alabama at Birmingham Institutional Review Board, the Jefferson County Department of Health (JCDH) in Birmingham, AL, USA, and the University of New Mexico Health Sciences Center Human Research Review Committee. Participants were enrolled from three populations: (1) predominantly African-American *C. trachomatis*-infected women aged over 16 years presenting to the JCDH STD Clinic in Birmingham, AL, USA; (2) Hispanic and non-Hispanic white virgin women aged 18–40 years presenting for routine gynecologic examinations in Albuquerque, NM, USA; and (3) men aged 18 and older reporting a history of urogenital Ct infection or no previous sexual contact (virgin) volunteering in Albuquerque, NM, USA. For population 1, Ct infection was confirmed by nucleic acid amplification testing (Hologic Aptima Combo 2; Hologic, Inc., Marlborough, MA, USA) and exclusion criteria included known co-infection with gonorrhea, syphilis, or HIV, as well as pelvic inflammatory disease. From all cohorts, serum was obtained from whole blood samples, which was used in ELISAs to measure IgG titers to the FxxN 1.1 peptide.

### 2.7. Statistical Analysis

Statistical analysis was performed utilizing GraphPad Prism 8 for macOS (Graphpad Software Inc., La Jolla, CA, USA). Normal Gaussian distribution was determined. If distribution was normal, one-way ANOVA without matching was utilized for statistical analysis. If distribution was not normal or sample size was insufficient, a nonparametric Kruskal–Wallis test was utilized.

## 3. Results

### 3.1. Rational Selection of Tetrapeptide Motif Epitopes for Vaccine Display

When determining an ideal tetrapeptide motif peptide candidate to display on the surface of Qβ VLPs, we considered the amino acid length needed to incorporate at least three tetrapeptide motifs, the location of the tetrapeptide motifs, predicted β-turns, B cell epitope predictions, the amino acids that are most commonly incorporated within FxxN motifs, and the amino acid conservation among urogenital Ct serovars. As is characteristic of all Ct Pmps, PmpD has multiple tetrapeptide motifs (FxxN and GGA(I,L,V)) located primarily within the passenger domain (Figure 1A). PmpD has approximately 40% more FxxN motifs than the average Ct Pmp, with 19 FxxN motifs. There are also 17 GGA(I,L,V) motifs present in PmpD, with only one located outside of the passenger domain. We utilized the Immune Epitope Database Antibody Epitope Prediction tool to determine predicted tetrapeptide B cell epitopes [36]. The majority of the PmpD passenger domain, encompassing most tetrapeptide motifs, was predicted to be a B cell epitope (AA 108–1193, Supplemental Figure S1). Additionally, data from our previous investigation of immunodominant epitopes in women with a history of urogenital Ct infection did not yield any clear FXXN or GGA(I,L,V) motifs of interest [35]. We prioritized AA 270–294 (FxxN 1, AA 270–294) as an epitope for display on Qβ VLPs (Figure 1A). The 270–294 FxxN/GGA(I,L,V) peptide has strong amino acid conservation among all urogenital Ct serovars and Cm, which we hypothesized could provide cross-protection and aid in the in vivo testing of our hypothesis (Figure 1B).

### 3.2. Engineering Qβ Virus-like Particle Vaccines Displaying the Tetrapeptide Motifs

**Figure 2:**
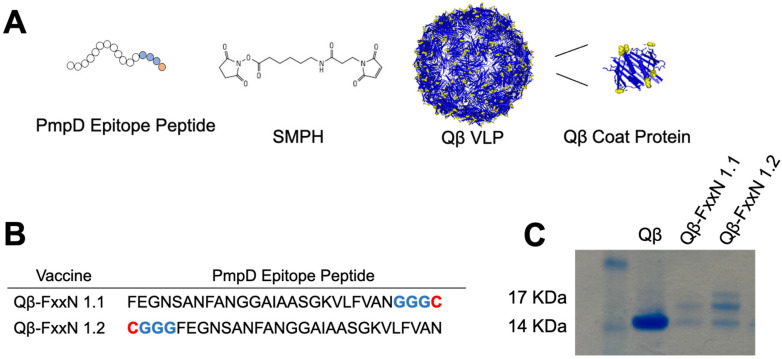
Chemical conjugation of tetrapeptide motifs onto the surface of Qβ VLPs. (**A**) 180 Qβ coat proteins self-assemble to form the Qβ VLP. Commercially purchased tetrapeptide motifs were chemically conjugated onto the surface of Qβ VLPs using the bifunctional crosslinker SMPH. (**B**) Vaccine naming strategy: C-terminal conjugation of the peptide is annotated “1.1” and N-terminal conjugation of the peptide is annotated “1.2”. Modifications are shown in blue and red, with terminal tri-glycine linker sequences and terminal cysteines added to facilitate conjugation. (**C**) Denaturing SDS PAGE showing successful conjugation of FxxN 1.1 and FxxN 1.2.

Using the highly immunogenic, recombinantly expressed bacteriophage Qβ VLPs as a display platform, we chemically conjugated our peptides of interest to surface exposed lysines on the VLPs using a bifunctional crosslinker (Figure 2A). The 270–294 FxxN/GGA(I,L,V) peptide (Figure 1B) was modified for chemical conjugation to Qβ on either the N- or C-terminus (Figure 2B). These peptides will hereafter be referred to as FxxN 1.1 (N-terminus conjugation) and FxxN 1.2 (C-terminus conjugation). Both peptides were successfully conjugated to the Qβ VLPs, as measured by denaturing SDS PAGE showing distinct laddering when the Qβ coat protein had peptide attached to the surface (Figure 2C).

### 3.3. Immunogenicity of Qβ-FxxN 1.1. and Qβ-FxxN 1.2 Alone and in Combination

**Figure 3:**
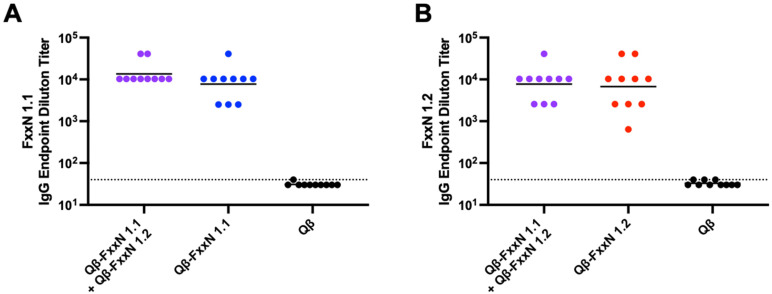
Immunization with Qβ VLP vaccines targeting tetrapeptide motifs result in high-titer IgG antibodies to cognate peptide. Endpoint IgG serum titers generated by cognate peptide antigens was measured for each vaccine formulation or Qβ control using peptide ELISA. (**A**) ELISAs were conducted with sera from mice immunized with Qβ-FxxN 1.1, the combined vaccine (Qβ-FxxN 1.1 + Qβ-FxxN 1.2) and Qβ against the FxxN 1.1 peptide. (**B**) ELISAs were conducted with sera from mice immunized with Qβ-FxxN 1.2, the combined vaccine (Qβ-FxxN 1.1 + Qβ-FxxN 1.2) and Qβ against the FxxN 1.2 peptide.

Female BALB/c mice (n = 10) were immunized with Qβ-FxxN 1.1, Qβ-FxxN 1.2 (alone or in combination), or Qβ (control) three times without the addition of exogenous adjuvant and assessed 3 weeks after final immunization for serum antibodies to cognate peptide(s). Mice showed IgG endpoint dilution titers of >10^4^ for most mice. The combination of Qβ-FxxN 1.1 and Qβ-FxxN 1.2 in a single immunization formulation did not impact overall endpoint dilution titer to either immunogen (Figure 3).

### 3.4. Immunization with Qβ-FxxN 1.1 + 1.2 Results in Protection from Urogenital Cm Infection in Mice

**Figure 4:**
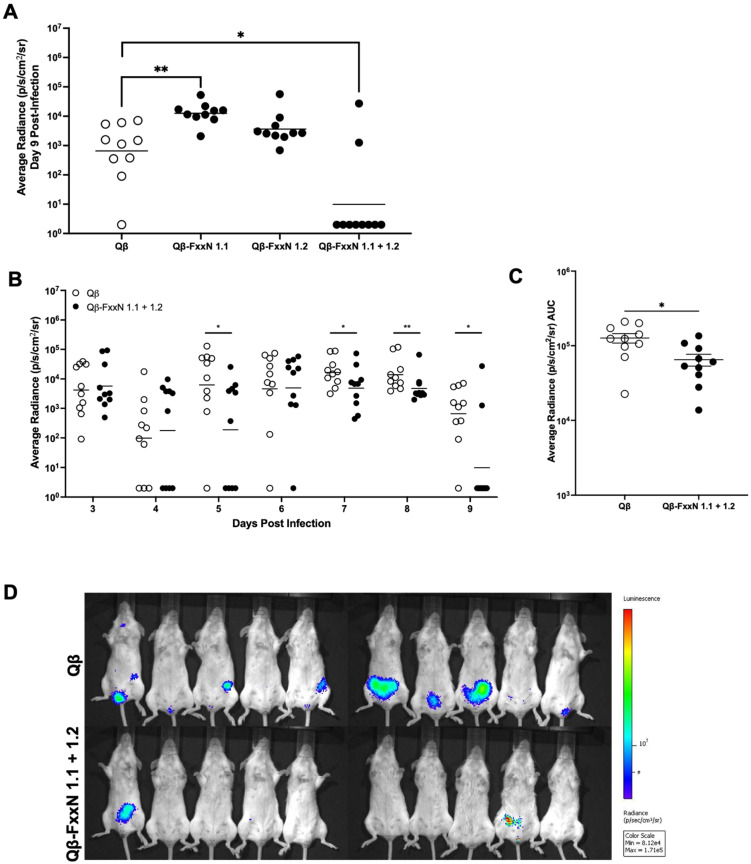
Immunization with Qβ-FxxN 1.1 + 1.2 results in protection from vaginal chlamydia infection. (**A**) Bacterial burden, as measured by average radiance, at day 9 post-infection for all vaccine groups, as compared to Qβ control. Only Qβ-FxxN 1.1 + 1.2 resulted in a statistically significant decreased bacterial burden at this time point, and 8 of 10 mice had undetectable chlamydia infection. (**B**) Time course of infection for mice immunized with Qβ-FxxN 1.1 + 1.2 and Qβ, over days 3 through 9 post-infection. (**C**) Average radiance area under the curve measured for Qβ-FxxN 1.1 + 1.2 and Qβ for all days investigated, resulting in a cumulative 0.29 log decrease in mean bacterial burden. (**D**) IVIS image of Qβ-FxxN 1.1 + 1.2 and Qβ control mice on day 9 post-infection. Statistical analysis was performed utilizing nonparametric Mann-Whitney *t*-test. Quantitative data represents the mean ± SEM. * *p* < 0.05, ** *p* < 0.01.

Having demonstrated that Qβ-FxxN 1.1 and Qβ-FxxN 1.2 immune sera IgG antibodies bind to cognate peptides, we next assessed our vaccines in a female urogenital mouse model of chlamydia infection that uses luciferase-expressing Cm [31]. Female BALB/c mice (n = 10/group) were immunized with Qβ-FxxN 1.1, Qβ-FxxN 1.2, Qβ-FxxN 1.1 + 1.2 or unconjugated Qβ (control). Mice received three intramuscular immunizations, three weeks apart, before being challenged vaginally with luciferase-expressing *Chlamydia muridarium* (Luc-Cm) (Figure 4A) [31]. Bacterial burden, as measured by average radiance, was monitored over days 3 through 9 post-infection via an in vivo imaging system (IVIS) (Figure 4A). Although Qβ-FxxN 1.1 or Qβ-FxxN 1.2 immunization alone did not result in a statistically significant decrease in Cm infection, combining these two peptide vaccines into one formulation provided a statistically significant decrease in the mouse vaginal Luc-Cm challenge model (Figure 4). Together, this resulted in a 0.29 log reduction in mean bacterial burden over all dates measured (Figure 4C). However, by day 9 post-infection, 8 out of 10 Qβ-FxxN 1.1 + 1.2 immunized mice had no detectable Cm infection compared to the control, where 9/10 mice vaccinated with unconjugated Qβ VLP (control) showed infection (Figure 4A).

### 3.5. Tetrapeptide Motifs Antigen Is a Non-Immunogenic Epitope in Natural Human Infection

**Figure 5:**
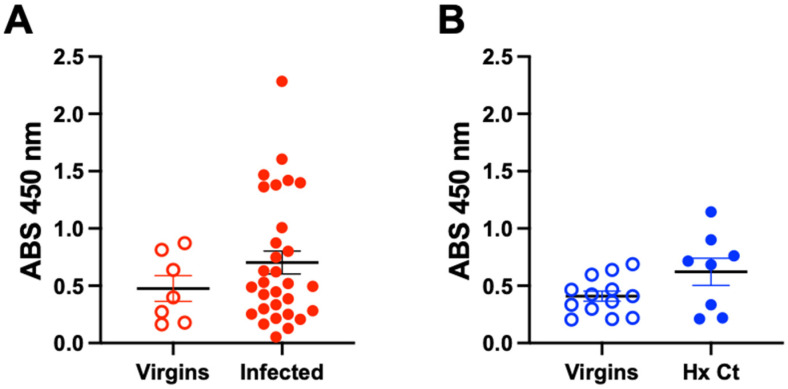
Reactivity of human sera to FxxN 1.1 peptide. Serum from women (**A**) and men (**B**) with active or a history of urogenital Ct infection. Sera were assessed for IgG to FxxN 1.1 peptide by ELISA using a 1:1400 dilution of sera. No statistical difference was observed between sexually naïve or Ct groups (active or history of Ct infection) for either men or women (women *p* value = 0.4351, men *p* value = 0.1253 by Mann-Whitney test).

Previous studies in our lab have finely mapped B cell epitopes of 24 Ct antigens, including PmpD, using deep sequence-coupled biopanning [35]. However, examination of these data showed that antibody responses to FxxN 1.1/1.2 were only present in 5/30 women with acute Ct infection, suggesting that this peptide is not immunogenic during natural infection [35]. To confirm this, we performed ELISA with serum from women with acute urogenital Ct infection (n = 30) and sexually naïve women (n = 7, controls) (Figure 5A). We saw no difference in antibody binding to FxxN 1.1 between sexually naïve or Ct-infected women. We also investigated serum from men with a history of Ct (n = 8) or sexually naïve men (n = 13). We also observed no difference in antibody binding to FxxN 1.1 in this population (Figure 5B).

## 4. Discussion

The tetrapeptide motifs of Ct Pmps are understudied yet promising vaccine targets due to their role in adhesion and ubiquitous nature within Ct serovars [15,16,20]. In this work, we rationally designed vaccine candidates targeting the tetrapeptide motifs of PmpD, hypothesizing that targeting repetitive motifs in this protein may elicit protective antibodies. We utilized a bacteriophage virus-like particle vaccine platform to display said tetrapeptide motifs at high valency on the surface of Qβ VLPs. Two Qβ-FxxN vaccine candidates produced peptide-specific antibody titers when delivered alone or in combination. We found that vaccination with individual peptides was unsuccessful at protecting against vaginal chlamydia challenge, but the combination vaccine (Qβ-FxxN 1.1 + 1.2) was able to mediate a 0.29 log reduction in mean bacterial burden over all days investigated, with 80% of mice completely clearing their infection by day 9 post-infection. Since FxxN 1.1 and 1.2 peptides only differ by the terminus on which they are conjugated to Qβ-VLPs, we hypothesize that immunization with a mixture of both Qβ-FxxN 1.1 and Qβ-FxxN 1.2 vaccines elicited a broader range of antibody specificities than immunization with either single-peptide VLP.

Other researchers have investigated PmpD as a vaccine using full-length recombinant PmpD or displaying the passenger domain of PmpD on various immunogenic vaccine delivery systems [24,26,27]. We observed an increased incidence of undetectable infection by day 9 with our mixed vaccine, with 8/10 animals having no detectable Luc-Cm by IVIS. Because the detection of infection requires the entry of Luc-Cm into cells and the translation of luciferase, this animal model is likely to be sensitive for detecting active infection in mice [31]. However, as with all methods of detecting infection, the limit of detection does not allow us to definitively indicate that clearance of infection has occurred. It is important to note that a limitation of our study is that we only conducted our animal challenge studies to 9 days post-inoculation. This limits our ability to make strong conclusions regarding the clearance of infection. A future study examining a longer time course of infection would provide more clarity on the overall impact of vaccination on infection. It will be important to test this vaccine in additional *Chlamydia trachomatis* models in order to assess protection against the human pathogen, and using cell infection assays as a detection method will likely increase the sensitivity. We observed a statistically significant increase in infection at day 9 for animals immunized with Qβ-FxxN 1.1 alone. This was surprising, and it is unclear what the importance of this finding is. Future studies should investigate these vaccines at later timepoints to understand the full scope of protection provided.

Data from our previously reported deep sequence-coupled biopanning of sera from women with a history of Ct infection indicated that women do not produce antibody responses toward FxxN 1.1/1.2 [35]. Additionally, we confirm by peptide ELISA in Figure 5 that women and men with either active infection or a history of infection do not generate strong antibodies responses, if any, to the FxxN 1.1/1.2 peptide epitope. This, coupled with our mouse challenge data showing that antibodies to this peptide reduce early infection, suggest that this FxxN 1.1/1.2 peptide epitope may be what some have called a “cryptic epitope”. A cryptic epitope is an epitope that is not normally immunogenic during natural infection but can be the target of protective antibody responses if those antibody responses are induced by a vaccine. Indeed, targeting cryptic epitopes has been explored for providing protection in other infectious diseases, including human immunodeficiency virus, SARS-CoV-2, and group A streptococcus, is being explored [37,38].

Future studies should further characterize interactions between antibodies targeting the tetrapeptide motifs, mechanisms of tetrapeptide antibody protection, and tetrapeptide motif antibody responses toward specific human Ct serovars. Pmp tissue and host tropisms may play a role in vaccine protection, and elucidating further vaccine targets for specific human disease will be essential. In this study, we show that Qβ-FxxN 1.1 + 1.2 is a promising Ct vaccine candidate, providing high-titer antibody responses that may bind to all urogenital Ct serovars due to the conserved nature of the epitope, and provides protection against urogenital chlamydia infection in a mouse model.

## 5. Conclusions

Our data support the conclusion that immunization with Qβ-FxxN 1.1 + 1.2 elicits high-titer antibody responses that can reduce the early infection of the lower reproductive tract of mice challenged with a luciferase-expressing *Chlamydia muridarum*.

## Data Availability

Data are either presented in this study or are available upon request by e-mailing the corresponding author.

## Supplementary Materials

The following supporting information can be downloaded at: https://www.mdpi.com/article/10.3390/vaccines13030234/s1, Figure S1: B cell epitope prediction, showing that the majority of the PmpD protein and passenger domain (AA 108–1193) is predicted to be surface-exposed and a possible B cell epitope (Immune Epitope Database Antibody Epitope Prediction Tool); Figure S2: Qβ-FxxN 1.1 and Qβ-FxxN 1.2.
