# Supplementary material for: Immunization Against Chlamydia trachomatis Polymorphic Membrane Protein D Tetrapeptide Motifs Limits Early Female Reproductive Tract Infection in a Mouse Model
Source: Vaccines (Basel). 2025 Feb 25;13(3):234. doi: 10.3390/vaccines13030234 (PMC11946637; doi:10.3390/vaccines13030234)
Supplement: Supplementary file 1 [file vaccines-13-00234-s001.zip › vaccines-3392707-supplementary.pdf]

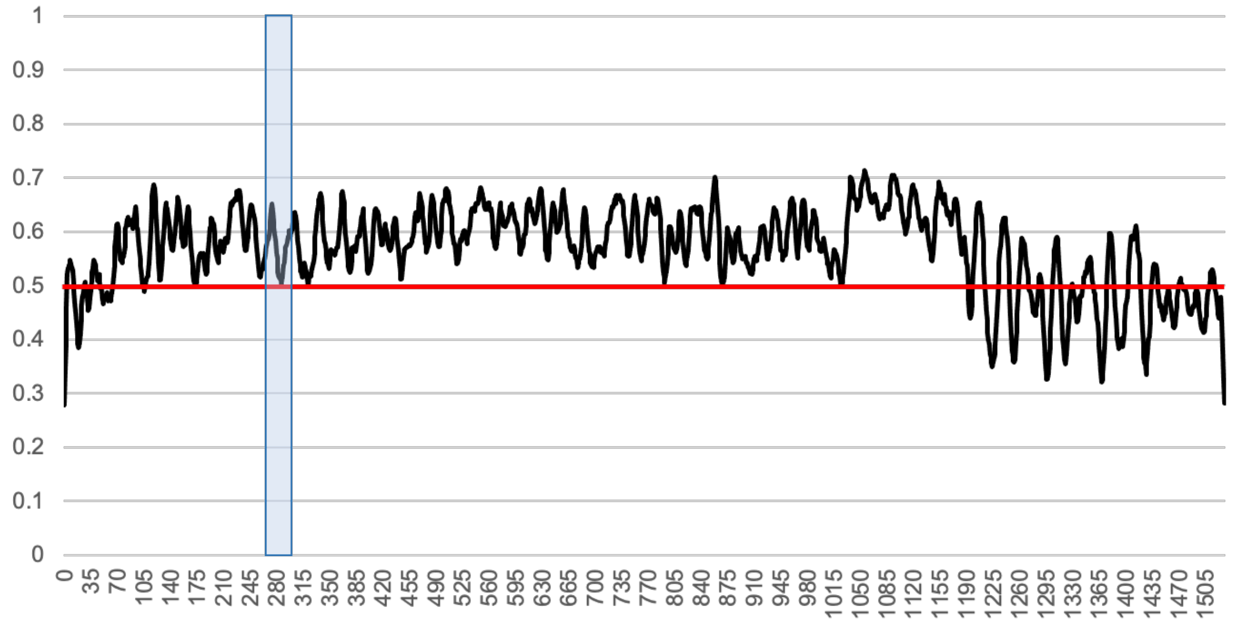

Supplementary Figure S1. B cell epitope prediction, showing that the majority of the PmpD protein and passenger domain (AA 108-1193) is predicted to be surface-exposed and a possible B cell epitope (Immune Epitope Database Antibody Epitope Prediction Tool). FxxN 1 location is shown in blue box.

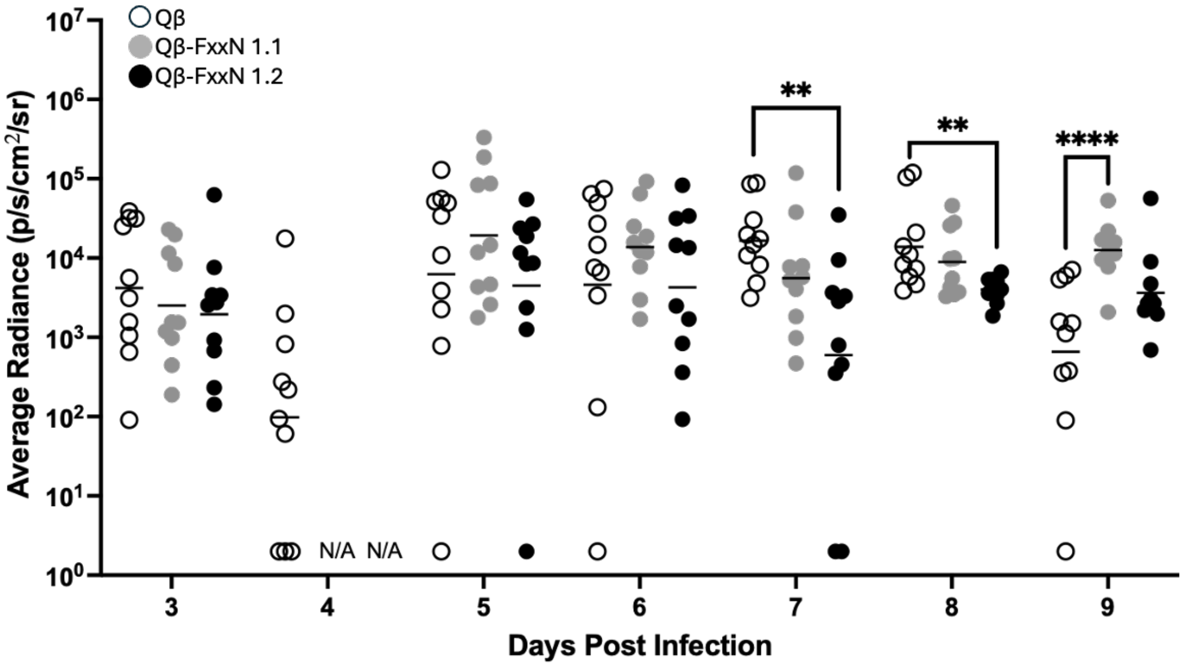

Supplementary Figure S2. Qβ-FxxN 1.1 and Qβ-FxxN 1.2. Time course of infection for mice immunized with Qβ-FxxN 1.1 or Qβ-FxxN 1.2, over days 3 through 9 post-infection. Statistical analysis was performed utilizing nonparametric Mann-Whitney t-test. Quantitative data represents the geometric mean. Qβ control is the same as shown in Figure 4.
